# Validation of collateral scoring on flat-detector multiphase CT angiography in patients with acute ischemic stroke

**DOI:** 10.1371/journal.pone.0202592

**Published:** 2018-08-24

**Authors:** Ilko L. Maier, Fabien Scalzo, Johanna R. Leyhe, Katharina Schregel, Daniel Behme, Ioannis Tsogkas, Marios-Nikos Psychogios, David S. Liebeskind

**Affiliations:** 1 Department of Neurology, University Medical Center Göttingen, Göttingen, Germany; 2 Neurovascular Imaging Res Core, Los Angeles, CA, United States of America; 3 Department of Neuroradiology, University Medical Center Göttingen, Göttingen, Germany; Universitatsklinikum Freiburg, GERMANY

## Abstract

**Background:**

The pivotal impact of collateral circulation on outcomes in endovascular therapy has fueled the development of numerous CTA collateral scales, yet synchronized validation with conventional angiography has never occurred. We validated multiphase flat-detector CTA (mpFDCTA) for collateral imaging in patients undergoing endovascular stroke treatment.

**Materials and methods:**

Consecutive acute ischemic stroke patient data, including mpFDCTA shortly followed by digital subtraction angiography (DSA), in the setting of acute ICA- or MCA-occlusions were analyzed. An independent core lab scored mpFDCTA with an established collateral scale and separately graded American Society of Interventional and Therapeutic Neuroradiology (ASITN) collateral score on DSA, blind to all other data.

**Results:**

24 consecutive cases (age 76.7 ± 7.3 years; 58.3% women; baseline NIHSS median 17 (4–23)) of acute ICA- or MCA-occlusion were analyzed. Time from mpFDCTA to intracranial DSA was 23.04 ± 7.6 minutes. Median mpFDCTA collateral score was 3 (0–5) and median DSA ASITN collateral score was 2 (0–3), including the full range of potential collateral grades. mpFDCTA and ASITN collateral score were strongly correlated (r = 0.86, p<0.001). mpFDCTA provided more complete collateral data compared to selective DSA injections in cases of ICA-occlusion. ROC analyses for prediction of clinical outcomes revealed an AUC of 0.76 for mpFDCTA- and 0.70 for DSA ASITN collaterals.

**Conclusions:**

mpFDCTA in the angiography suite provides a validated measure of collaterals, offering distinct advantages over conventional angiography. Direct patient transfer to the angiography suite and mpFDCTA collateral grading provides a novel and reliable triage paradigm for acute ischemic stroke.

## Introduction

Numerous studies proved evidence for the superiority of endovascular therapy (EVT) in combination with intravenous thrombolysis (IVT) compared to IVT alone in patients with acute large vessel occlusion (LVO) of the anterior cerebral circulation [1]. These positive results were not only driven by improved endovascular devices and peri-procedural strategies, but also by a refinement of patient selection criteria, including the use of collateral [2] and perfusion status [3].

In this respect, collateral status plays a central role for prognostication and patient selection in everyday clinical practice. Numerous studies showed that collateral status, both quantified by using CTA- and DSA-derived collateral scores, predicts successful revascularization, fate of ischemic tissue and favorable functional outcome in patients with EVT [4–9].

DSA-derived collateral status according to the American Society of Interventional and Therapeutic Neuroradiology (ASITN) collateral grading system hereby is the most commonly used and validated way to determine collaterals [10], assessing anatomic and dynamic features of collateral flow with high spatial and temporal resolution. This scale has been recently compared to pre-EVT multidetector CTA (MDCTA) collateral scores showing equal predictive performance [11].

However, synchronized validation with multiphase flat-detector CTA (mpFDCTA) has not yet been done. This validation is crucial concerning the newly proposed one-stop management of acute stroke patients, which has been shown to significantly reduce door to groin time by omitting MDCT and performing acute stroke imaging in the angiography suite directly prior to MT [12].

The aim of our study was to compare the diagnostic and prognostic accuracy of collateral status determined on mpFDCTA with the gold standard of collateral status determination on DSA to provide further evidence for the reliability of multimodal FDCT-derived stroke imaging in patients treated with the novel one-stop approach in acute major ischemic stroke.

## Material and methods

### Patient selection

Data was analyzed from a prospectively derived, monocentric database including peri- and post-procedural neuroimaging and neurological data of consecutive patients being treated according to the one-stop paradigm of acute major stroke treatment. This approach has been described in detail elsewhere [12–14]. In brief, it includes a streamlined treatment of acute stroke patients with a National Institutes of Health Stroke Scale score (NIHSS) of ≥10 (in-house practice between 2015–2017) or ≥7 (in-house practice since 2017) with direct angio suite referral, bypassing MDCT and using noncontrast FDCT and FDCTA instead. In case of LVO-diagnosis on FDCTA, patients were directly treated with mechanical thrombectomy. IVT was administered after the exclusion of an intracerebral hemorrhage (ICH) as per current guidelines (symptom to needle times ≤ 4,5 h and exclusion of other contraindications; bridging IVT).

Clinical data including stroke scores (NIHSS and modified Rankin Scale (mRS)) were determined by a certified stroke neurologist. Favorable functional outcome was defined as a mRS ≤ 2 at 90 days or at discharge, if 90 days data was missing.

Ethics approval was sought from the ethics committee of the University Medicine Göttingen and all patients or next of kin gave informed written consent for the anonymized use of disease-related data on hospitalization.

### Imaging acquisition

FDCT was acquired with a biplane flat-detector angiography system (Artis Q; Siemens Healthcare, Forchheim, Germany). For FDCT, following parameters were used: 20 s rotation; 200° total angle with ~500 projections; 109 kV; 1.8 μGy/frame; effective dose ~2.5 mSv. Then, a biphasic FDCTA (biFDCTA) was acquired for detection of arterial occlusion. For the biFDCTA, 60 mL contrast agent (Imeron 400; Bracco Imaging, Konstanz, Germany) were administered intravenously at an injection rate of 5 mL/s, followed by 60 mL saline chaser at the same injection rate. For the biFDCTA, the following imaging parameters were used: 2 x 10s rotation; 200° total angle, 0.8°/frame angulation step; 70kV, 1.2 μGy/frame, effective dose ~ 2.5 mSv. The first rotation was timed after a bolus watching DSA to capture the peak arterial phase, while the second phase is acquired automatically after a 5 s delay to depict the venous phase. Both FDCTA datasets were instantly and automatically reconstructed and 24 mm transversal maximal intensity projections of the first and second phase were simultaneously viewable on a commercially available workstation (Syngo X Workplace; Siemens). Raw FDCT and DSA data were extracted from the department’s picture archiving and communication system, anonymized and sent to an external core-lab for collateral evaluation (D.L.).

### Imaging evaluation

Collaterals status were judged according to a modified multiphase collateral score on mpFDCTA [12] and according to the American Society of Interventional and Therapeutic Neuroradiology/Society of Interventional Radiology (ASITN/SIR) Collateral Flow Grading System on DSA [15]. The modified multiphase collateral score compares the collaterals within the occluded vascular territory to the asymptomatic contralateral hemisphere and ranges from 0 (no vessels visible) to 5 (no delay and normal/increased prominence/extent of pial vessels). The ASITN collateral score ranges from 0 (no collaterals visible to the ischemic site) to 4 (complete and rapid collateral blood flow to the vascular bed in the entire ischemic territory by retrograde perfusion). For the latter, “N/A” is scored if another territory than the MCA territory is affected or if contrast injection for collateral status is not possible. Early ischemic signs were determined using the Alberta Stroke Program Early CT Scale (ASPECTS) [16] on non-contrast FDCT and follow up non-contrast MDCT. An experienced reader determined collateral status with both scores with a 4-week break, in order to limit recall bias.

### Statistical analysis

Statistical analysis was performed using MedCalc statistical package (MedCalc 16.8; MedCalc Software bvba, Ostend, Belgium). Characteristics of all patients are shown as mean ± standard deviation (SD), if normally distributed, and as median with interquartile range (IQR), if not.

Collateral status was compared using the Pearson correlation. To assess the predictive value of the modified collateral score and ASITN/SIR collateral scale for clinical outcome and decrease in ASPECTS, area under the receiver operator characteristic curve (AUROC) analysis has been used. Adjustment of ROC-curves for measured covariables was considered, but after investigations in our dataset no meaningful association between covariables, e.g. age or mTICI-value to the marker could be found. Following [17], no further adjustment was being made. The comparison of ROC-curves was done using the paired DeLong test, cut-off scores were defined as scores with maximal Youden-Index. For all statistical methods, P-values below 0.05 were considered significant.

## Results

Collateral status was determined on mpFDCTA and DSA in 24 patients with a mean age of 77 (± 7) years, a median NIHSS of 17 (IQR, 13–19) and a median ASPECTS of 8 (IQR, 7–9) points at baseline (Table 1). From these patients, 18 (75%) had an M1-occlusion, followed by 5 (20.8%) patients with a carotid terminus- and 1 (4.2%) with an M2-occlusion. Recanalization was successful in 22 (91.7%) of patients with a mean symptom-to-reperfusion time of 195 ± 66 min and a mean FDCTA-to-first-intracranial DSA of 23.04 ± 7.6 min. Conscious sedation was performed in 14 (58.3%) patients and only 2 (8.3%) received general anesthesia. There was no use of sedatives in the remaining 8 (33.3%) patients.

**Table 1:** Baseline characteristics.

Parameter (n = 24)	
Age (mean ± SD)	76.7 ± 7.3
Sex (male, %)	10 (41.7)
NIHSS at baseline (median, IQR)	17 (13–19)
ASPECTS at baseline (median, IQR)	8 (7–9)
Occluded vessel	
Carotid terminus (n, %)	5 (20.8)
MCA (M1) (n, %)	18 (75)
MCA (M2) (n, %)	1 (4.2)
Successful reperfusion (n, %)	22 (91.7)
Time metrics	
Symptom to FDCT (mean min ± SD)	145 ± 70
Symptom to reperfusion time (n = 22, mean ± SD)	195 ± 66
Door to groin (mean min ± SD)	26 ± 7
Groin to reperfusion (n = 22, mean ± SD)	37 ± 20
FDCT to first DSA (min ± SD)	23 ± 8
Ship	
Mothership (n, %)	12 (50)
Drip and ship (n, %)	7 (29.2)
Ship and drip (n, %)	1 (4.2)
Just ship (n, %)	4 (16.7)
Wake up stroke (n, %)	4 (16.7)
IVT (n, %)	16 (66.7)
Any anesthesia (n, %)	14 (58.3)
General anesthesia at the time of FDCT (n, %)	2 (8.3)
Clinical characteristics	
Arterial Hypertension (n, %)	18 (75)
Hyperlipoproteinaemia (n, %)	10 (43.5)
Diabetes mellitus (n, %)	8 (33.3)
Atrial fibrillation (n, %)	15 (65.2)
Coronary artery disease (n, %)	8 (33.3)
Chronic kidney failure (n, %)	6 (25)

IQR: interquartile range; SD: Standard deviation; MCA M1/M2: Medial cerebral artery in its M1 or M2 segment; IVT: intravenous thrombolysis; FDCT: flat-detector computed tomography; DSA: Digital subtraction angiography; NIHSS: National Institute of Health Stroke Scale; mRS: modified Rankin Scale; ASPECTS: Alberta Stroke Program Early CT Scale

As shown in Table 2, median modified collateral score on mpFDCTA was 3 (2–4) and median ASITN/SIR collateral score was 2 (2–3) on DSA, including the full range of potential collateral grades. In two out of five patients with carotid terminus occlusion, collaterals could not be determined by selective DSA injections compared to a complete visualization of collaterals in all patients using mpFDCTA (imaging of one of these cases is shown in online S1 Fig).

**Table 2:** Multiphase flat-detector CTA (mpFDCTA) collateral score and American Society of Interventional and Therapeutic Neuroradiology (ASITN) collateral grading score on DSA (n = 24).

mpFDCTA			DSA		
collateral score	number of patients (n, %)	median score (IQR)	collateral score	number of patients (n, %)	median score (IQR)
0	1 (4.2)	3 (2–4)	0	2 (8.3)	2 (2–3)
1	1 (4.2)		1	3 (12.5)	
2	6 (25)		2	10 (41.7)	
3	8 (33.3)		3	7 (29.2)	
4	7 (29.2)		4	0 (0)	
5	1 (4.2)		N/A	2 (8.3)	

IQR: interquartile range; N/A: not applicable

**Fig 1:**
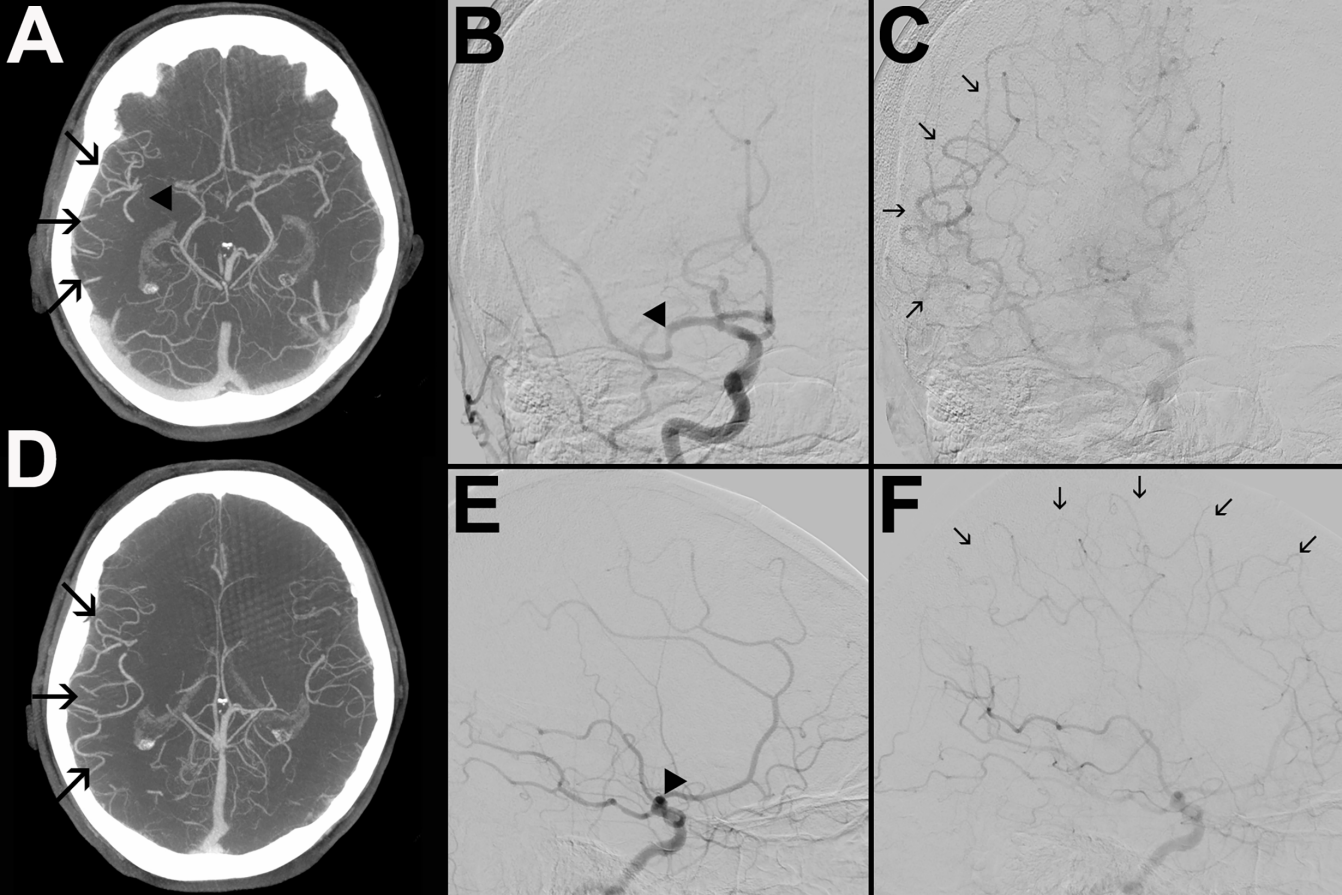
mpFDCTA (A, D) and DSA images in posterior-anterior (B, C) and lateral projection (E, F) of a patient with good collaterals are shown. B and E depict an early and C and F a later phase of the angiogram. The arrowheads (A, B, E) indicate an occluded M1-segment of the right MCA. On mpFDCTA, the prominence of pial vessels within the right hemisphere is increased (A, D). Angiographic collateral flow of the ischemic bed is slow, but complete (arrows in C and F). Hence, the patient was assigned collateral scores of 5 and 3 on mpFDCTA and DSA, respectively.

**Fig 2:**
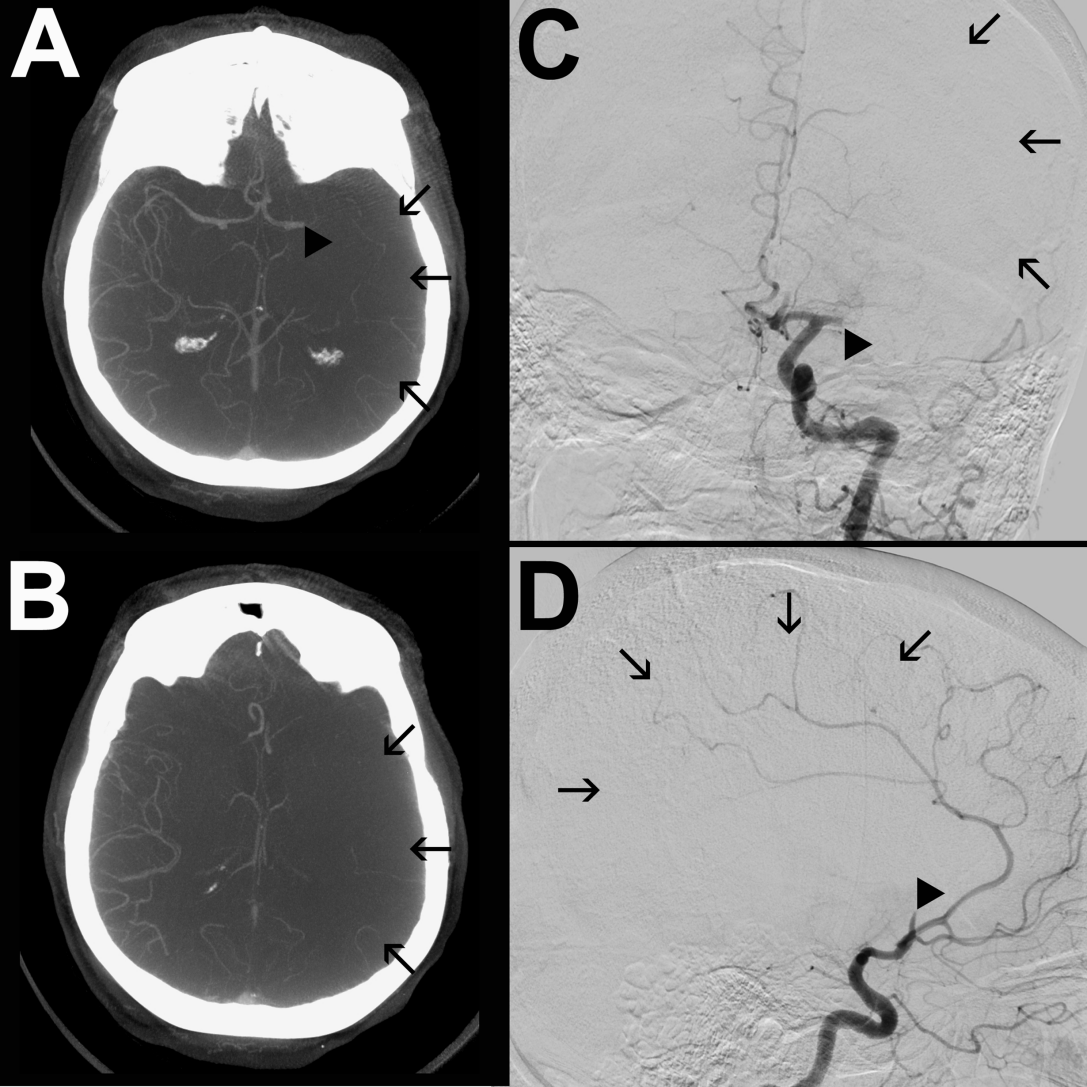
mpFDCTA (A, B) and DSA images in posterior-anterior (C) and lateral projection (D) of a patient with poor collaterals are shown. The arrowheads (A, C) indicate an occluded M1-segment of the left MCA. There are no collaterals identifiable in the ischemic hemisphere. The arrows (A-D) indicate the ischemic bed with missing collateral vessels. Thus, the patient was assigned a collateral score of 0 on both, mpFDCTA and ASITN DSA score.

Figs 1 and 2 show representative cases of one patient with poor- and one with good collateral status both on mpFDCTA and DSA. There was a strong correlation between collateral scores on mpFDCTA and DSA (r = 0.86, p<0.001; see online S2 Fig).

From the 24 included patients, 10 (41.6%) had a favorable functional outcome with a follow up mRS ≤ 2. These patients had significantly higher median modified collateral score on mpFDCTA- and tended to have higher ASITN/SIR collateral scores on DSA (3.5 (3–4) vs. 3 (2–3), p = 0.013, and 2 (2–3) vs. 2 (1–2.25), p = 0.080, respectively). Predictive value for favorable functional outcome of both collateral scores were moderate (modified collateral score on mpFDCTA: AUC 0.796, p<0.001; ASITN/SIR collateral score on DSA: AUC: 0.714, p = 0.034; see online S3 Fig). The modified collateral score on mpFDCTA had a higher predictive value compared to the ASITN/SIR collateral score (p = 0.028). Cut-off scores for favorable outcome of modified collateral score on mpFDCTA was 2 with a positive predictive value (PPV) of 63% and a negative predictive value of 100%. The Cut-off of ASITN/SIR collateral score on DSA was 1 with a PPV of 54% and NPV of 100% (see online S1 Table).

Patients with a decrease of ≤ 2 points of the ASPECTS score from baseline to 24 h follow-up non-contrast MDCT had a significant higher mpFDCTA- and ASITN/SIR collateral score on DSA (3.5 (3–4) vs. 2 (1.5–3), p = 0.007, and 2 (2–3) vs. 2 (1–2), p = 0.032, respectively). Predictive value for ASPECTS-decay ≤ 2 points of modified collateral score on mpFDCTA was good (AUC 0.836, p<0.001) and moderate for ASITN/SIR collateral score on DSA (AUC: 0.776, p = 0.002; see online S4 Fig). Again, the comparison of predictive value of both scores revealed no significant difference (p = 0.129). Cut-off scores for ASPECTS-decay of modified collateral score on mpFDCTA was 3 and of ASITN/SIR collateral score on DSA 2 (see online S2 Table).

## Discussion

This pilot study validates collateral status on mpFDCTA as a unique imaging technique by comparing it to the reference standard of DSA. We provide evidence for the reliability of mpFDCTA to judge collateral status in the context of the one-stop approach in acute stroke treatment, in which collateral status can be relevant for patient selection and is crucial for prognostication.

The one-stop paradigm for EVT-eligible patients, which is an innovative approach to avoid in-house time delays and associated decreasing chances of favorable outcomes [18, 19], includes multimodal stroke imaging and treatment in the angio suite [12]. However, using this approach, modern angiography systems must be capable to answer following questions with high diagnostic accuracy: 1) Is there an ICH? 2) What is the extend of non-salvageable ischemic tissue? 3) Is there an interventionally treatable LVO? 4) What is the status of proximal arterial vessels? And 5) Is there (and what is the extend of the) infarct core? For question 1 and 2, there is growing evidence that latest generation FDCTs are able to reliably exclude ICH [20, 21] as well as can reliably be used for the determination of ASPECTS [21, 22].

Concerning question 3 and 4, our study, together with findings from Yang et al. (already demonstrating the feasibility of collateral status evaluation with FDCTA [23]), provides further evidence for the use of FDCTA in the one-stop management paradigm in acute ischemic stroke. We could demonstrate, that this method has equal predictive value compared to DSA derived collateral status evaluation. Besides the time saving aspect, mpFDCTA has distinct advantages compared to DSA: for full collateral evaluation, contrast agent application of multiple arteries is necessary using DSA, whereas mpFDCTA provides a global view of collaterals with a single IV injection rather than by a series of intra-arterial injections. In this respect, to judge collateral status in patients with carotid terminus occlusions on DSA, a pan-angiography is necessary, which leads to time delays. In our study, collaterals in two patients with carotid terminus occlusion could not be determined with DSA because of the aforementioned problem, which demonstrates the ability of mpFDCTA to provide more complete information on collateral status in patients treated with EVT. Moreover, using mpFDCTA provides complete information on occlusions or stenosis of proximal vessels, which is important for acute decision making (e.g. angioplasty/stenting vs. conservative treatment in cases with contralateral occlusion/stenosis of the internal carotid artery).

Concerning the last question (question five), it has already been demonstrated that collateral status is a surrogate marker for the infarct core extend [24]. By validating mpFDCTA collateral status against the reference modality DSA, we provide evidence for the use of mpFDCTA collateral status to predict infarct core.

Taking all these findings into consideration, FDCT-imaging in patients eligible for EVT seems to be feasible, provides reliable information about collateral status and has distinct advantages compared to the imaging strategy preferred in most stroke centers to date. Determining collateral status on mpFDCTA has time saving advantages compared to usual MDCTA and procedural advantages compared to DSA, as outlined above. Using mpFDCTA to diagnose LVO, to investigate the status of proximal vessels and collateral status might therefore contribute to increased favorable outcome in acute stroke patients when applied in a larger scale.

Limitations of our study include the retrospective, single center design and the judgement of collateral scores by one experienced rater only. The small sample size also limits the generalizability of our results, which are likely to be reproducible only if similar or more advanced FDCT systems and imaging reconstruction methods are being used. Moreover, the results can not be directly related to current thrombectomy-trials including collateral status by using triphasic protocols (e.g. the ESCAPE trial) and not directly related to conventional CTA, being predominantly used to judge collateral status to date. In this study, a 3-vessel pan-angiography prior thrombectomy was not performed to be able to achieve reperfusion as fast as possible. Therefore, ASITN/SIR collateral score was judged on the first diagnostic DSA of the target vessel territory and might have neglected pial collaterals from the contralateral hemisphere or the posterior circulation. This might also have resulted in the higher predictive value of the modified collateral score on mpFDCTA for clinical outcome, as it might have visualized pial collaterals from other territories better compared to DSA. Although patients included in this study received FDCT-imaging under the use of an improved head holder with better head fixation and eye covering [21] the scan period of 2x10 sec of the biphasic FDCTA can result in severe motion artifacts, which represents another technical limitation.

In conclusion, our study provides evidence for the diagnostic reliability of mpFDCTA derived collateral status compared to the well validated determination of collateral status on DSA using the ASITN/SIR collateral grading system. In addition, using mpFDCTA in the one-stop paradigm of acute stroke treatment could result in better functional outcome of EVT-eligible patients in the future, especially concerning the reduction of door-to-groin times by skipping MDCT and avoiding the need to visualize multiple vessels with angiography to achieve complete status of cerebral collateralization [25].

## Supporting information

S1 Fig mpFDCTA (A, B) and DSA images in posterior-anterior (C) and lateral projection (D) of a patient with an occluded right carotid terminus (arrowheads in A, C, D) are shown. A moderate reduction of prominence and extent of pial vessels within the right hemisphere can be seen on mpFDCTA (B). Hence, a collateral score of 3 was assigned. In contrast, it is not possible to evaluate the collateral status on angiograms (C, D) derived from contrast-injection in the terminally occluded ICA. For this purpose, a panangiography is necessary, but was not performed in this case. This demonstrates a procedural advantage of mpFDCTA compared to DSA, as full collateral evaluation is possible with just a single i.v. contrast injection and one image acquisition.
(PDF)

S2 Fig Pearson correlation between multiphase flat-detector CTA and ASITN collateral score on DSA. (PDF)

S3 Fig Area under the receiver operator curve analysis for the predictive value of multiphase flat-detector CTA and ASITN collateral score on DSA for functional outcome. (PDF)

S4 Fig Area under the receiver operator curve analysis for the predictive value of multiphase flat-detector CTA and ASITN collateral score on DSA for ASPECTS decay of ≥ 2 points. (PDF)

S1 Table Cut-off scores with classification functions of mpFDCTA- and ASITN-DSA collateral scores for favorable functional outcome. (PDF)

S2 Table Cut-off scores with classification functions of mpFDCTA- and ASITN-DSA collateral scores for a decrease of ≤ 2 points of the ASPECTS from baseline to routine 24 h non-contrast CT. (PDF)
