# Supplementary material for: Validation of collateral scoring on flat-detector multiphase CT angiography in patients with acute ischemic stroke
Source: PLoS One. 2018 Aug 24;13(8):e0202592. doi: 10.1371/journal.pone.0202592 (PMC6108461; doi:10.1371/journal.pone.0202592)
Supplement: S1 Fig — mpFDCTA (A, B) and DSA images in posterior-anterior (C) and lateral projection (D) of a patient with an occluded right carotid terminus (arrowheads in A, C, D) are shown. A moderate reduction of prominence and extent of pial vessels within the right hemisphere can be seen on mpFDCTA (B). Hence, a collateral score of 3 was assigned. In contrast, it is not possible to evaluate the collateral status on angiograms (C, D) derived from contrast-injection in the terminally occluded ICA. For this purpose, a panangiography is necessary, but was not performed in this case. This demonstrates a procedural advantage of mpFDCTA compared to DSA, as full collateral evaluation is possible with just a single i.v. contrast injection and one image acquisition. (PDF) [file pone.0202592.s001.pdf]

Data Supplement

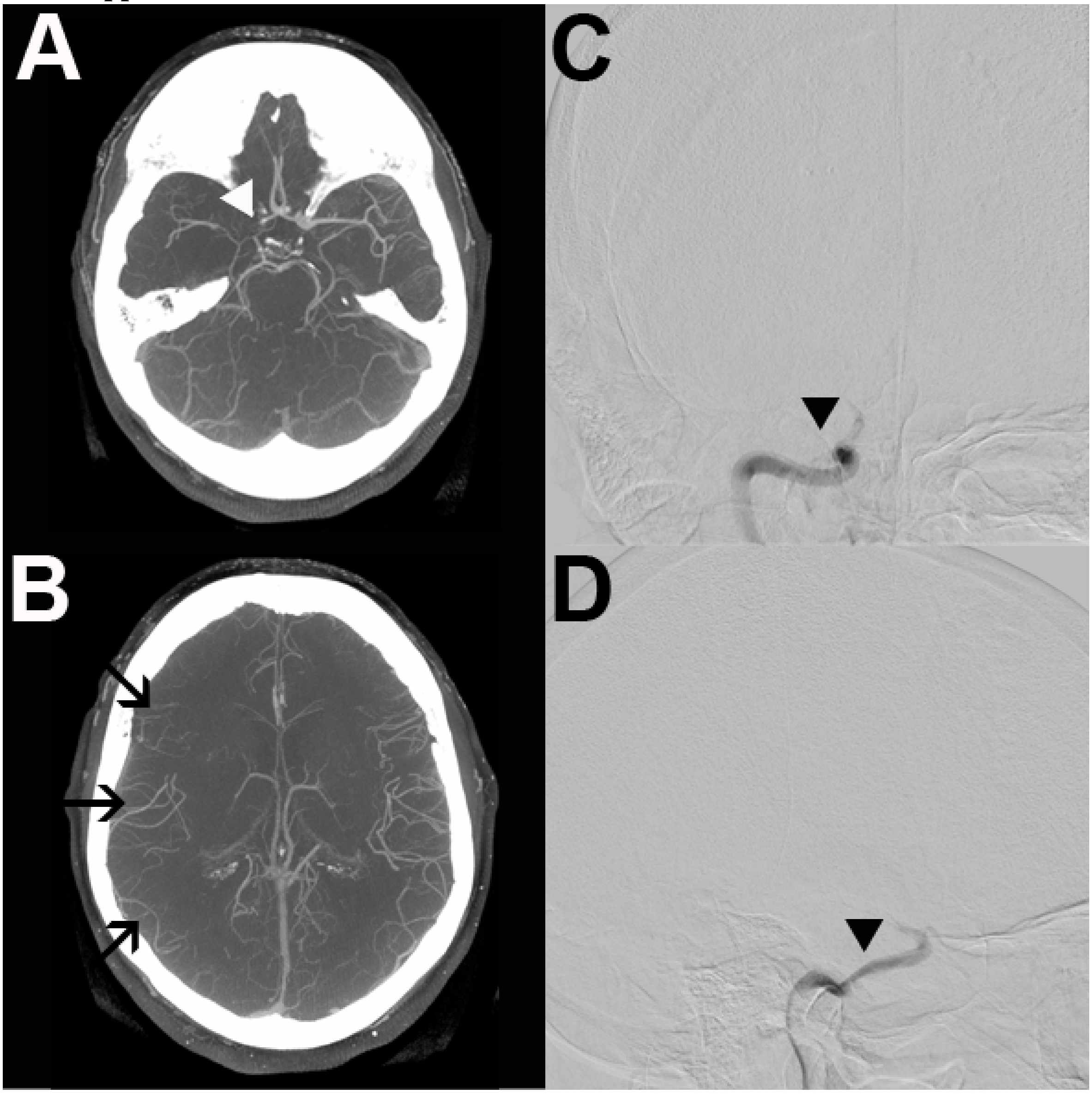

S1 Fig: mpFDCTA (A, B) and DSA images in posterior-anterior (C) and lateral projection (D) of a patient with an occluded right carotid terminus (arrowheads in A, C, D) are shown. A moderate reduction of prominence and extent of pial vessels within the right hemisphere can be seen on mpFDCTA (B). Hence, a collateral score of 3 was assigned. In contrast, it is not possible to evaluate the collateral status on angiograms (C, D) derived from contrast-injection in the terminally occluded ICA. For this purpose, a panangiography is necessary, but was not performed in this case. This demonstrates a procedural advantage of mpFDCTA compared to DSA, as full collateral evaluation is possible with just a single i.v. contrast injection and one image acquisition.
