# Supplementary material for: Validation of collateral scoring on flat-detector multiphase CT angiography in patients with acute ischemic stroke
Source: PLoS One. 2018 Aug 24;13(8):e0202592. doi: 10.1371/journal.pone.0202592 (PMC6108461; doi:10.1371/journal.pone.0202592)
Supplement: S2 Fig — (PDF) [file pone.0202592.s002.pdf]

Data Supplement

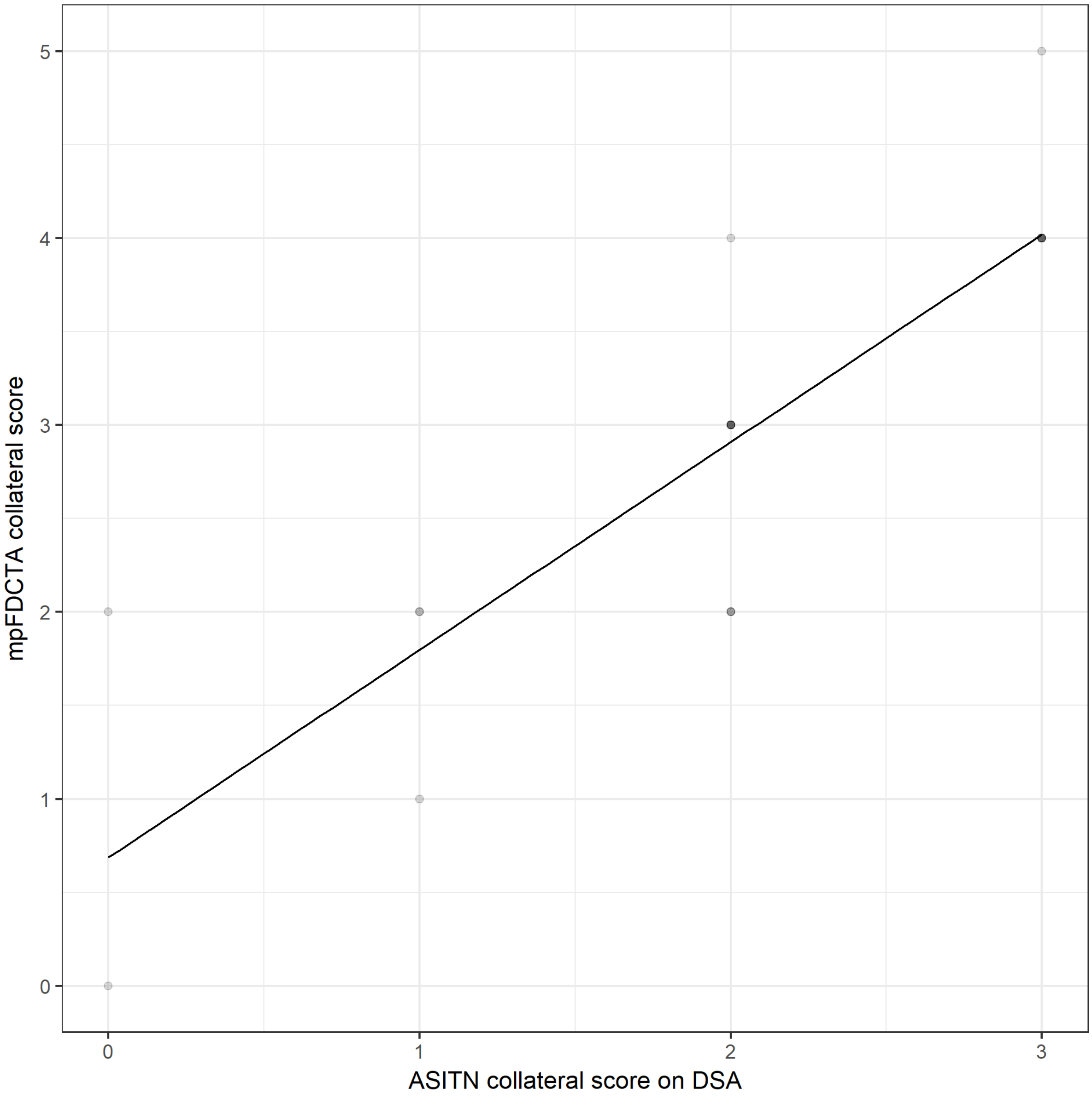

S2 Fig: Pearson correlation between multiphase flat-detector CTA and ASITN collateral score on DSA.
