# Supplementary material for: Validation of collateral scoring on flat-detector multiphase CT angiography in patients with acute ischemic stroke
Source: PLoS One. 2018 Aug 24;13(8):e0202592. doi: 10.1371/journal.pone.0202592 (PMC6108461; doi:10.1371/journal.pone.0202592)
Supplement: S4 Fig — (PDF) [file pone.0202592.s004.pdf]

Data Supplement

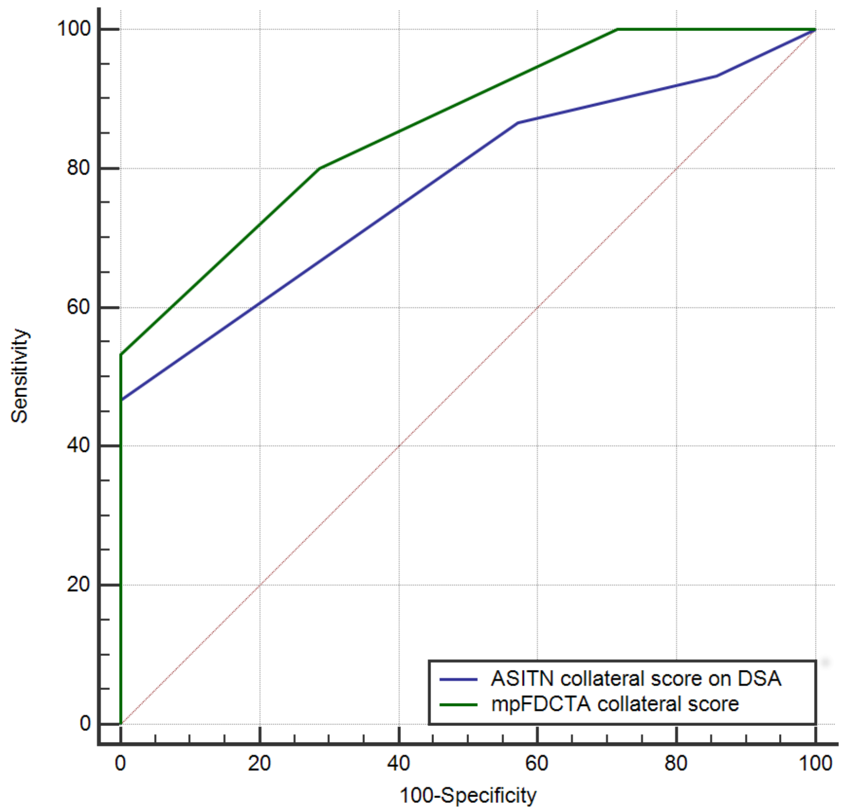

S4 Fig: Area under the receiver operator curve analysis for the predictive value of multiphase flat-detector CTA and ASITN collateral score on DSA for ASPECTS decay of ≥ 2 points.
