# Supplementary material for: Validation of collateral scoring on flat-detector multiphase CT angiography in patients with acute ischemic stroke
Source: PLoS One. 2018 Aug 24;13(8):e0202592. doi: 10.1371/journal.pone.0202592 (PMC6108461; doi:10.1371/journal.pone.0202592)
Supplement: S1 Table — (PDF) [file pone.0202592.s005.pdf]

Data Supplement

S1 Table: Cut-off scores with classification functions of mpFDCTA- and ASITN-DSA collateral scores for favorable functional outcome

Cut-off score	Favorable functional outcome (mRS ≤ 2)							
	Sensitivity	95% CI	Specificity	95% CI	PPV	95% CI	NPV	95% CI
mpFDCTA collateral score								
> 1	100	69.2-100	14.29	1.8-42.8	45.7	41.6-40.9	100	n.a.
> 2*	100	69.2-100	57.14	28.9-82.3	62.74	48.9-74.8	100	n.a.
> 3	50	18.7-81.3	78.57	49.2-95.3	63.29	29.2-87.8	68.57	54.3-80
ASITN-DSA collateral score								
> 0	100	66.4-100	15.38	1.9-45.4	46	41.5-50.6	100	n.a.
> 1*	100	66.4-100	38.46	13.9-68.4	53.87	44.6-62.9	100	n.a.
> 2	44.44	13.7-78.8	76.92	46.2-95	58.08	24.1-85.8	65.5	50.9-77.7

mpFDCTA: multiphase flat-detector computed tomography angiography; ASITN: American Society of Interventional and Therapeutic Neuroradiology; DSA: digital subtraction angiography; mRS: modified Rankin Scale; PPV: positive predictive value; NPV: negative predictive value; CI: confidence interval; n.a.: not applicable
