# Supplementary material for: Validation of collateral scoring on flat-detector multiphase CT angiography in patients with acute ischemic stroke
Source: PLoS One. 2018 Aug 24;13(8):e0202592. doi: 10.1371/journal.pone.0202592 (PMC6108461; doi:10.1371/journal.pone.0202592)
Supplement: S2 Table — (PDF) [file pone.0202592.s006.pdf]

Data Supplement

S2 Table: Cut-off scores with classification functions of mpFDCTA- and ASITN-DSA collateral scores for a decrease of ≤ 2 points of the ASPECTS from baseline to routine 24 h non-contrast CT

Cut-off score	ASPECTS-decrease ≤ 2 points from baseline to 24 h ncCT							
	Sensitivity	95% CI	Specificity	95% CI	PPV	95% CI	NPV	95% CI
mpFDCTA collateral score								
> 2	81	54-96	63	25-92	90	68-98	4	16-76
> 3*	50	25-75	100	63-100	n.a.	n.a.	n.a.	n.a.
> 4	6,25	0-30	100	63-100	n.a.	n.a.	n.a.	n.a.
ASITN-DSA collateral score								
> 1	87	60-98	43	10-82	83	67-92	50	14-86
> 2*	47	21-73	100	59-100	n.a.	n.a.	n.a.	n.a.
> 3	0	0-22	100	59-100	n.a.	n.a.	n.a.	n.a.

mpFDCTA: multiphase flat-detector computed tomography angiography; ASITN: American Society of Interventional and Therapeutic Neuroradiology; DSA: digital subtraction angiography; ASPECTS: Alberta Stroke Program Early CT Scale; ncCT: Non-contrast computed tomography; CI: confidence interval; PPV: positive predictive value; NPV: negative predictive value; n.a.: not applicable
